# Hyperhomocysteinemia and pregnancy outcomes in women with polycystic ovary syndrome: A case-control study

**DOI:** 10.18502/ijrm.v21i2.12807

**Published:** 2023-03-08

**Authors:** Elene Asanidze, Jenaro Kristesashvili, Nino Parunashvili, Manana Urjumelashvili, Zurab Tsetskhladze, Aleksandre Asanidze

**Affiliations:** ^1^Department of Medical Faculty of Teaching University Geomedi, Tbilisi, Georgia.; ^2^Department of Medical Faculty of Ivane Javakhishvili Tbilisi State University, Tbilisi, Georgia.; ^3^University of Toronto, Faculty of Medicine, Physician Assistant Program, Toronto, Canada.; ^4^Teaching University Geomedi, Tbilisi, Georgia.; ^5^Tbilisi State Medical University, Tbilisi, Georgia.

**Keywords:** Polycystic ovary syndrome, Hyperhomocysteinemia, Recurrent abortion, Insulin resistance.

## Abstract

**Background:**

One of the reproductive medicine challenges is to determine the role of hyperhomocysteinemia in the pathogenesis of polycystic ovary syndrome (PCOS), especially in women with recurrent pregnancy loss (RPL).

**Objective:**

Determine the correlation between hyperhomocysteinemia and pregnancy outcome in women with PCOS.

**Materials and Methods:**

This case-control study involved 245 women (20-30 yr) and was conducted in Georgia, Tbilisi from 2019-2022. Of these, 175 were women with PCOS (study group) and 70 were healthy women (control group). Women with PCOS were divided into group I with RPL (n = 90), and group II with live births (n = 85). Group I was divided into subgroups A and B with and without insulin resistance. The investigation measured homocysteine (Hcy), follicle-stimulating, luteinizing, anti-Mullerian hormones, total and free testosterone were determined. To determine the ovarian volume and antral follicle count, participants also underwent an ultrasound examination.

**Results:**

In women with PCOS, the average Hcy level was significantly higher than in the controls, p < 0.05. In group I, the average Hcy level was significantly higher than in group II and controls, p < 0.05. There was no significant difference in average Hcy level between group II and controls. The average Hcy level in group I, subgroup A was significantly higher than in subgroup B, p < 0.05. The average total, free testosterone levels, and homeostatic model assessment-insulin resistance levels (HOMA-IR) in group I was significantly higher than in group II and controls. HOMA-IR in group II and controls did not differ significantly. The average anti-Mullerian hormone levels in women with PCOS were significantly higher than controls, p < 0.05. No significant difference was observed in average anti-Mullerian hormone level, ovarian volume, antral follicle count, and body mass index between the comparison groups of PCOS. In group I, a positive correlation between Hcy with HOMA-IR was detected.

**Conclusion:**

Serum Hcy levels are elevated in women with PCOS and RPL, which correlates with their insulin resistance status.

## 1. Introduction

Polycystic ovary syndrome (PCOS) is one of the most common endocrinopathies associated with metabolic derailments and complex disorders involving reproductive and cardiovascular systems (1, 2).

In cases of PCOS, multiple individuals or combined factors lead to fertility issues (3, 4). PCOS is one of the most important risk factors not only for infertility but also for spontaneous abortion. According to various studies, the frequency range of miscarriages in PCOS have been reported to range from 25-73% (5, 6).

Among several hypotheses, endocrine and metabolic disorders of the syndrome, like insulin resistance (IR), obesity, and hyperandrogenemia, have been implicated as risk factors for reproductive failure (7-10).

Hyperhomocysteinemia (Hhcy) has been considered an independent risk factor for developing several diseases, including cardiovascular disease, arterial and venous thrombosis, neuropsychogenic diseases, and adverse pregnancy outcomes (11, 12). It has been established that Hhcy increases the hypercoagulable state of pregnancy and the likelihood of developing thrombosis in the maternal-fetal circulatory system and, as a result, the undesirable outcome of pregnancy (11-14). In the early stages of pregnancy, including the preimplantation period, the development of pregnancy loss is facilitated by Hhcy due to the disruption of blood flow in the endometrial blood vessels through implantation disorders. Hence, implantation disorders may explain “preclinical" pregnancy loss and infertility. In the later stages of pregnancy, Hhcy promotes the development of complications such as vascular dysfunction, hypertensive disorders, preeclampsia, preterm labor, preterm placental abruption, intrauterine growth retardation, and fetal death (13, 15, 16).

There are controversial opinions regarding the link between Hhcy and pregnancy outcomes in women with PCOS. Some studies show that spontaneous abortions in women with PCOS are associated with Hhcy, and it was suggested that it might be considered one of the key features of PCOS, so it is vital to know the association between elevated homocysteine (Hcy) levels and miscarriages in women with PCOS (3, 13, 17). Furthermore, a few studies show a possible association between IR and Hhcy in PCOS women with recurrent pregnancy loss (RPL), but some studies did not find a significant difference in Hcy levels among PCOS women with RPL and PCOS women without pregnancy complications (16, 18, 19).

There is limited evidence that Hcy plays a role in the pathogenesis of PCOS. There are only a small number of studies done so far, with insufficient material, inconsistent results, and vague inclusion for recommendations in patients' management based on Hcy levels. This is a strong indicator that further studies are required in this direction.

This study aimed to determine the link between Hhcy and pregnancy outcome in women of reproductive age with PCOS and investigate the relationship between the elevated level of Hcy and IR, as well as obesity and hormonal and morphological characteristics of the ovary in PCOS women.

## 2. Materials and Methods 

The case-control study involved 245 Georgian women (20-30 yr). Of which, 175 women were with PCOS (study group) and 70 were age-matched healthy women (control group). This study was conducted at the Center for Reproductive Medicine `Universe' (Georgia, Tbilisi) from January, 2019 to March, 2022.

The diagnosis of PCOS was based on the criteria of the 2003 Rotterdam Consensus (1). Women with PCOS (Study group), based on reproductive anamnesis, were divided into 2 groups:

• Group I- 90 women, experienced RPL.

• Group II- 85 women with live births in anamnesis.

Control group- healthy women without a personal and family history of PCOS, miscarriages, and pregnancy complications.

PCOS women with RPL, based on homeostatic model assessment-insulin resistance levels (HOMA-IR), were divided into 2 subgroups: subgroup A with insulin resistance, HOMA-IR ≥ 2.5 (n = 48) and subgroup B without insulin resistance, HOMA-IR < 2.5 (n = 42).

RPL is defined as 2 or more clinical pregnancy losses (20).

### Criteria for inclusion in the study

All the participants did not take a drug containing sex hormones 6 months before inclusion in the study. A hormonal investigation was performed for all participants between 2-3 days from their menstrual cycle. The investigation measured Hcy, follicle-stimulating hormone, luteinizing hormone (LH), anti-Mullerian hormone (AMH), total (T) and free testosterone (FT) were determined. To determine the ovarian volume (Ov/v) and antral follicle count, participants also underwent an ultrasound examination (Voluson E10) at this time. Hcy was measured using Hcy enzyme immunoassay.

HOMA-IR was calculated using the formula: fasting insulin (microU/L) x fasting glucose (nmol/L)/ 22.5. Body mass index (BMI), free androgen index (FAI) and sex hormone binding globulin (SHBG) were calculated. Hirsutism was evaluated on the Ferriman-Gallwey modified scale (mFG).

We compared group I (women with PCOS and RPL) and group II (women with PCOS and live births in anamnesis) with controls to investigate the association between Hhcy and pregnancy outcome in women of reproductive age with PCOS. For the investigation of the relationship between the elevated level of Hcy and IR, as well as obesity, hormonal, and morphological characteristics of the ovary in PCOS women, we compared these 2 groups of PCOS (group I and group II) with each other.

### Ethical considerations

All the participants in the study were informed in advance, the essence and goal of the study were explained, and written consent for their participation in the study was obtained. The Ethics Committee of the `Center for Reproductive Medicine Universe' agreed to conduct the study. Approval code: 11/22; Tbilisi, Georgia.

### Statistical analysis

Based on the linear regression between the variables, we used Pearson's correlation coefficient r to define the dependence of Hcy on the HOMA-IR in the patients studied. We used the One-Way ANOVA test to compare hormonal, clinical, and ultrasound parameters between the groups of PCOS. The methodology used in our study follows the most applied guidance for statistical analysis in the medical sciences (21). To simplify statistics and increase the perceptibility of the tables providing the statistical analysis results, we expressed the level of variability of the analyzed variables by showing the value of their standard deviation. Statistical analysis was done using office software MS Excel 2021 and Statistical Package for the Social Sciences, version 24.0 (SPSS 24.0, Chicago, IL, USA). A p-value of 0.05 or lower is considered statistically significant.

## 3. Results

The mean age of women with PCOS (study group) was 26.8 ± 3.4 and the mean age of control group was 25.2 ± 3.9 yr. No statistical difference was observed between groups regarding age.

**Figure 1:**
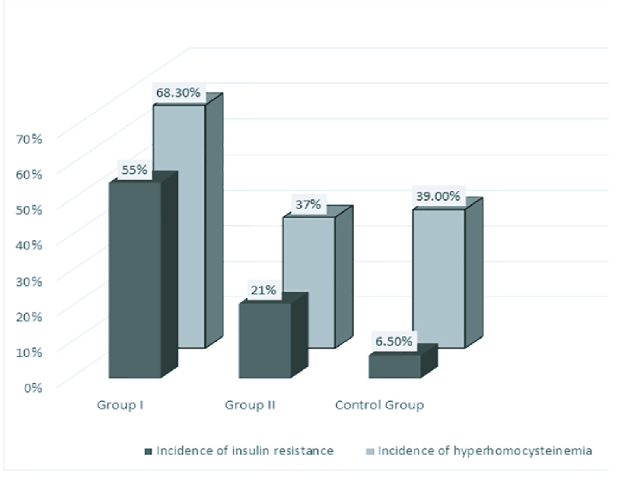
Distribution of women according to the incidence of hyperhomocysteinemia and insulin resistance.

In the study group, women with PCOS (group I and group II together) average Hcy levels were significantly higher than in controls (p < 0.05). Average Hcy level in group I was significantly higher than in group II and the control group (p < 0.05, Table I). No statistically significant difference was observed in average Hcy level between group II and controls (Table I). The average Hcy level in group I subgroup A (14.9 ± 3.1 µmol/L) was significantly higher than in group I subgroup B (9.5 ± 2.8 µmol/L), p < 0.05. Other hormonal and ovarian morphological characteristics in comparable subgroups did not differ significantly (in group I subgroup A average AMH-12.2 ± 3.2 ng/ml; T-1.38 ± 0.3 ng/ml; FT-3.4 ± 1.3 pg/ml; LH-9.4 ± 3.1 /IU/I; Ov/v 13.7 ± 6 cm³; AFC-32. In group I subgroup B AMH-11.4 ± 3.8 ng/ml; T-1.28 ± 0.3 ng/ml; FT-3 ± 1.2 pg/ml; LH-8.2 ± 2.6 /IU/I; Ov/v 12.1 ± 5.6 cm³; AFC-30). Incidence of Hhcy and insulin resistance in women with PCOS with RPL was 68.3% and 55%, respectively, which was significantly higher when compared to the group with live births (HHcy: 36%; IR:21%, p < 0.05) and controls (HHcy: 39%; IR: 6.5%) (Figure 1). The incidence of insulin resistance in PCOS women with live births (group II) was significantly higher than in controls, but the incidence of Hhcy in group II and control group did not differ significantly (Figure 1). HOMA-IR in group I was significantly higher than in group II and control group (p < 0.05). HOMA-IR in PCOS women with live births and controls did not differ significantly (Table I). There was no significant difference in average AMH and LH levels, ovarian volume, antral follicle count, and BMI between comparison groups of PCOS (Table I).

Average AMH levels in PCOS women (group I and group II-10.8 ± 2.8) were significantly higher compared to controls (2.9 ± 5), p < 0.05.

In group I, the average level of T, FT, FAI, SHBG, and Ferriman-Gallwey modified scale were significantly higher than in group II and controls (Table I).

**Figure 2:**
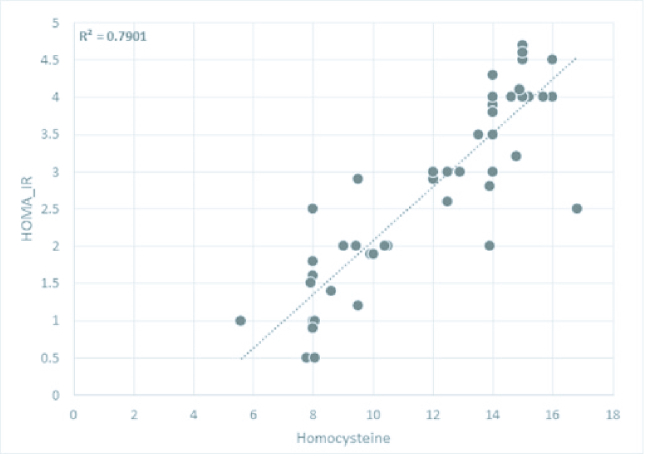
Correlation between homocysteine with homeostatic model assessment for insulin resistance (HOMA-IR) in group I (PCOS and RPL). We used Pearson's correlation coefficient `r' based on linear regression.

In group I statistically significant positive correlation between Hcy with HOMA-IR was detected (p < 0.05) (Figure 2).

In group I, subgroup A- a statistically significant positive correlation was found between Hcy and HOMA-IR, BMI, and OV/v (p < 0.05).

In group I, subgroup B- there was only a statistically significant positive correlation between Hcy and the Ov/v (p < 0.05).

In group II and control group, there was no correlation between Hcy with HOMA-IR and hormonal and ovarian morphological characteristics.

**Table 1:** Hormonal, clinical, and ultrasound (US) parameters of participants


**Parameter **	**Group I (PCOS and RPL)**	**Group II (PCOS and live born)**	**Control group**
**Homocysteine (µmol/L)**	12.24 ± 2.5* ${}^{\wedge }$	8.1 ± 2.2	7.2 ± 2.4
**AMH (ng/ml)**	11.8 ± 3.8 ${}^{\wedge }$	9.8 ± 1.8^□^	2.9 ± 1.5
**T (ng/ml)**	1.3 ± 0.3* ${}^{\wedge }$	0.3 ± 0.2^□^	0.3 ± 0.1
**FT (pg/ml)**	3.8 ± 1.8* ${}^{\wedge }$	2.1 ± 1.5^□^	1.9 ± 1.2
**FAI**	3.32*	0.4^□^	0.4
**SHBG (mm/l)**	39.9 ± 18.5* ${}^{\wedge }$	68 ± 22.5	71 ± 29.5
**LH /IU/I**	8.8 ± 2.5 ${}^{\wedge }$	7.9 ± 3.6^□^	5.5 ± 2.2
**HOMA-IR**	3.1 ± 2.1* ${}^{\wedge }$	1.8 ± 0.5	1.5 ± 0.2
**BMI (kg/m^2^)**	22.5 ± 4.5 ${}^{\wedge }$	21.2 ± 2.5	19.2 ± 3.5
**mFG**	18 ± 4.5* ${}^{\wedge }$	7.7 ± 3.3^□^	4.5 ± 2
**Ov/v (cm^3^)**	12.9 ± 6.5 ${}^{\wedge }$	11 ± 5.7^□^	6.8 ± 1.5
**AFC**	31 ± 11.5 ${}^{\wedge }$	29 ± 6.5^□^	8.3 ± 6.5
Data are presented as Mean ± SD, comparison of the data in group I and group II: *p < 0.05. Comparison of the data at group I and control group: ${}^{\wedge }$ p < 0.05. Comparison of the data at group II and control group: ^□^ p < 0.05. PCOS: Polycystic ovary syndrome, LH: Luteinizing hormone, AMH: Anti-Mullerian hormone, T: Total, FT: Free testosterone, Ov/v: Ovarian volume, AFC: Antral follicle count, HOMA-IR: Homeostatic model assessment for insulin resistance, BMI: Body mass index, FAI: Free androgen index, SHBG: Sex hormone binding globulin, mFG: Ferriman-Gallwey modified scale			

## 4. Discussion

PCOS is a multifactorial syndrome and is associated with important reproductive disorders. Several confounding factors involving in the pathogenesis of PCOS, individually or in combination, may contribute to developing thrombosis and lead to RPL (21). This study indicated a link between Hhcy and pregnancy outcomes in PCOS patients. Our study shows that average Hcy levels were significantly higher in PCOS patients who experienced RPL than in PCOS patients with a live birth in anamnesis and controls. According to our results, Hhcy may be considered as one of the causes of pregnancy loss in patients with PCOS. Our findings are consistent with the studies of a few other authors (14, 17, 22), but some studies did not find a significant difference in Hcy levels among PCOS women with RPL and without pregnancy complications (19, 23).

PCOS is commonly associated with metabolic and cardiovascular complications, and IR is the potential pathogenetic mechanism for both (22, 24, 25). Insulin resistance and compensatory hyperinsulinemia are one of the most important events in the pathogenesis of PCOS. Our investigation showed that the average HOMA-IR in PCOS patients with RPL was significantly higher compared with PCOS patients with live births and controls. Our study results showed that the insulin resistance index in PCOS patients with live births and controls did not differ significantly. A similar finding was confirmed by other studies (16, 19, 23). Other authors, however, did not find a significant association between insulin resistance and RPL in PCOS patients (26).

When we compared the groups of PCOS women, we found that in patients with RPL the average level of T, FT, and FAI were significantly higher than in the group with live births. There were positive correlations among serum Hcy, HOMA-IR, and androgen levels in PCOS women with RPL. Few studies found similar outcomes. They described that in PCOS women, compensatory hyperinsulinemia due to insulin resistance may lead to ovarian and adrenal androgen hypersecretion. Furthermore, there is an increased level of FT due to high insulin levels suppressing hepatic production of SHBG (26).

The relationship between insulin resistance status and Hhcy in PCOS women is still a topic of debate. Some studies found a positive correlation between Hcy and insulin resistance in PCOS women, which was related to thrombosis in the fetoplacental system and may be considered as a cause of pregnancy loss (2, 3, 5, 15, 18, 26). Others did not confirm this association and proposed that serum Hcy levels rise independently in PCOS women (17, 27). Our research indicated a relationship between Hhcy and insulin resistance in women with PCOS and RPL. According to our results, we detected a positive correlation between Hcy and insulin resistance in PCOS women with RPL. The results may indicate the importance of measuring the Hcy levels and insulin resistance status in women with PCOS for predicting RPL.

Our study results showed that in PCOS women with RPL and insulin resistance, average Hcy levels were significantly higher than in PCOS patients with RPL without insulin resistance, but other characteristics in comparable subgroups did not differ significantly. Similar findings were confirmed by other studies (28), however, some studies reported higher levels of AMH in PCOS women with insulin resistance, compared with those with normal insulin sensitivity (9, 29). In further contrast, some studies found a negative correlation between AMH and HOMA-IR has also been reported (14).

A strong relationship exists between serum Hcy level and insulin resistance status in women with PCOS and RPL, which contributes to the long-term complications of PCOS. However, larger sample sizes and randomized trials are needed to establish the role of Hhcy in women with PCOS and RPL and to rule out genetic factors of Hhcy.

Screening for Hcy status and correction of Hhcy in women with PCOS will help avoid pregnancy losses by preventing thrombosis in the maternal-fetal circulatory system and improving reproductive performance and pregnancy outcome.

## 5. Conclusion

Serum Hcy levels are elevated in women with PCOS and RPL, which correlates with their insulin resistance status. Screening for Hcy status and correction of Hhyc and insulin resistance in women with PCOS might improve reproductive outcomes.

##  Conflict of Interest

The authors declare that there is no conflict of interest. 
